# Supplementary material for: Sida chlorotic leaf virus: a new recombinant begomovirus found in non-cultivated plants and Cucumis sativus L
Source: PeerJ. 2023 Mar 22;11:e15047. doi: 10.7717/peerj.15047 (PMC10039651; doi:10.7717/peerj.15047)
Supplement: Supplemental Information 1 [file peerj-11-15047-s001.docx]

**Table S1.** List of primers used in this study for specific detection of component A of BGVs identified via Illumina sequensing.

| **Primer Name** | **Sequence** | **Target** |
| --- | --- | --- |
| F-Rep_Si | GAGCACTTCTTCCGTCGATC | Amplification of upper DNA-A region of SiChLV (the region between nucleotide 2139-929) |
| R-CP_Si | GCATACACAGGATTAGAGGCA |  |
| F-CP_Si | TGTCTATAACCACCAGGAAGC | Amplification of lower DNA-A region of SiChLV (the region between nucleotide 831-2155) |
| R-Rep_Si | CCAGATCGACGGAAGAAGTG |  |
| F-EuMV | CCCACTCTTGCATCTCTTCC | Fragment amplification of component A of EuMV |
| R-EuMV | GACTCCAGGACTCCACAAAC |  |
| F-SiMSinV | CGGATGTGAGGGTGATGAAG | Fragment amplification of component A of SiMSiV |
| R-SiMSinV | CGAACGATCCTACACAGTGAC |  |
| F-OYMMV | CTGGTCCTCGTGTACAATGG | Fragment amplification of component A of OYMMV |
| R-OYMMV | GATTCTTCGACCTGGTGTCC |  |
| F-CuChLV | AACGTCCTTGGATCACCGA | Fragment amplification of component A of CuChLV |
| R-CuChLV | GCAGTGCTAGGTTCATTGTC |  |
