# Supplementary material for: Sida chlorotic leaf virus: a new recombinant begomovirus found in non-cultivated plants and Cucumis sativus L
Source: PeerJ. 2023 Mar 22;11:e15047. doi: 10.7717/peerj.15047 (PMC10039651; doi:10.7717/peerj.15047)
Supplement: Supplemental Information 3 [file peerj-11-15047-s003.docx]

| **CuChLV vs SiChLV** | |
| --- | --- |
| **Region** | **Pairwise identity (%)** |
| CP ORF | 93.4 |
| REn ORF | 99.4 |
| TrAP ORF | 98.9 |
| AC4 ORF | 62 |
| REP ORF | 77 |
| IR | 79 |
| DNA-A | 86.2 |

**Table S3.** Genomic comparison between SiChLV and CuChLV
