# Supplementary material for: Sida chlorotic leaf virus: a new recombinant begomovirus found in non-cultivated plants and Cucumis sativus L
Source: PeerJ. 2023 Mar 22;11:e15047. doi: 10.7717/peerj.15047 (PMC10039651; doi:10.7717/peerj.15047)
Supplement: Supplemental Information 5 [file peerj-11-15047-s005.docx]

**Table S5.** Probable parents for recombination

| **Probable Parents** | **GenBank Accession Number** |
| --- | --- |
| Corchorus yellow spot virus | DQ875868.1 |
| Macroptilium golden mosaic virus-[Jamaica:Wissadula:August Town] | EU158096 |
| Tomato chlorotic leaf curl virus | KY449277.1 |
| Tomato mosaic Trujillo virus | KY449275.1 |
| Potato yellow mosaic virus | AY965897.1 |
| Cabbage leaf curl virus isolate Ecuador-El Oro | MH359390.1 |
| Squash mild leaf curl virus-[Imperial Valley] | AF421552.1 |
| Bean leaf crumple virus | KX857725.1 |
