# Supplementary material for: Sida chlorotic leaf virus: a new recombinant begomovirus found in non-cultivated plants and Cucumis sativus L
Source: PeerJ. 2023 Mar 22;11:e15047. doi: 10.7717/peerj.15047 (PMC10039651; doi:10.7717/peerj.15047)
Supplement: Supplemental Information 6 [file peerj-11-15047-s006.docx]

**Table S6.** Begomoviruses that infect plants from Sida genus and at least one plant from other genera.

| **BGVs** | **Natural host** | **Alternative host** | **GenBank ID** | **Reference** |
| --- | --- | --- | --- | --- |
| Chayote yellow mosaic virus | Sechium edule | Sida acuta | KT454826.1 | Unpublished |
| Cotton leaf curl Gezira virus | Gossypium spp. | Sida spp. | AY036007.1 | Virus Genes. 2002, Vol. 24(3), pp 249-56 |
| Okra yellow mosaic Mexico virus | Abelmoschus esculentus | Sida rhombifolia | GU990613 | Unpublished |
| Sida golden mosaic Honduras virus | Sida spp. | Hibiscus sabdariffa | KY767922.1 | Unpublished |
| Sida golden mosaic virus | Sida spp. | Phaseolus vulgaris (Bean) | GQ357649 | Plant Dis. 2010, Vol. 94(4), pp 487 |
| Sida micrantha mosaic virus | Sida spp. | Glycine max (Soybean) | FJ686693.1 | Arch Virol. 2009, Vol. 154(9), pp 1567-70 |
| Sida micrantha mosaic virus | Sida spp. | Phaseolus vulgaris (Bean) | HM357459.3 | Unpublished |
| Sida micrantha mosaic virus | Sida spp. | Solanum lycopersicum (Tomato) | KC706537.1 | J Virol. 2013, Vol. 87(10), pp 5784-99 |
| Sida mosaic Bolivia virus 2 | Sida spp. | Salvia hispanica(Chia) | KJ742421.1 | Unpublished |
| Sida mosaic Sinaloa virus | Sida spp. | Solanum lycopersicum (Tomato) | KX440612.1 | Unpublished |
| Sida mottle Alagoas virus | Sida spp. | Passiflora edulis f. flavicarpa (Maracuya) | MH371469.1 | Unpublished |
| Sida mottle virus | Sida spp. | Glycine max (Soybean) | AY436328.1 | Unpublished |
| Sida yellow blotch virus | Sida spp. | Phaseolus lunatus (Lima bean) | KX640991.1 | Unpublished |
| Sida yellow net virus | Sida spp. | Passiflora edulis f. flavicarpa (Maracuya) | MF957207 | Unpublished |
| Sida yellow net virus | Sida spp. | Solanum lycopersicum (Tomato) | KU996357.1 | Unpublished |
| Soybean chlorotic blotch virus | Glycine max | Sida cordifolia | KT454812.1 | Unpublished |
| Squash leaf curl virus | Cucurbita spp. | Sida spp. | KX620943.1 | Unpublished |
| Tobacco curly shoot virus | Nicotiana tabacum | Sida acuta | LC316184.1 | Unpublished |
| Tomato chlorotic mottle virus | Solanum lycopersicum | Sida spp. | KC706545.1 | J Virol. 2013, Vol. 87(10), pp 5784-99 |
| Tomato mild mosaic virus | Solanum lycopersicum | Sida urens | JX871375.1 | Unpublished |
| Tomato mild mosaic virus | Solanum lycopersicum | Sida urens | KC706609.1 | J Virol. 2013, Vol. 87(10), pp 5784-99 |
| Tomato severe rugose virus | Solanum lycopersicum | Sida spp. | JX865638.1 | J Gen Virol. 2013, Vol. 94(Pt 2), pp 418-31 |
| Tomato yellow distortion leaf virus | Solanum lycopersicum | Sida spp. | HE806450.1 | Virus Genes. 2014, Vol. 49(2), pp 312-24. |
| Tomato yellow spot virus | Solanum lycopersicum | Sida spp. | KX348226.1 | Unpublished |
