# Supplementary material for: Sida chlorotic leaf virus: a new recombinant begomovirus found in non-cultivated plants and Cucumis sativus L
Source: PeerJ. 2023 Mar 22;11:e15047. doi: 10.7717/peerj.15047 (PMC10039651; doi:10.7717/peerj.15047)
Supplement: Supplemental Information 7 [file peerj-11-15047-s007.docx]

**Table S7.** Begomoviruses that infect plants from Malvastrum genus and at least one plant from other genera.

| **BGVs** | **Natural host** | **Alternative host** | **GenBank ID** | **Reference** |
| --- | --- | --- | --- | --- |
| Tobacco leaf curl virus | Nicotiana tabacum | Malvastrum coromandelianum | FN397861.1 | Unpublished |
| Tobacco curly shoot virus | Nicotiana tabacum | Malvastrum coromandelianum | MF977705.1 | Unpublished |
| Papaya leaf curl virus | Carica papaya | Malvastrum coromandelianum | DQ359120.1 | Unpublished |
| Tomato yellow leaf curl China virus | Solanum lycopersicum | Malvastrum coromandelianum | KC189894 | Unpublished |
| Cotton leaf curl Bangalore virus | Gossypium spp. | Malvastrum coromandelianum | LC316185 | Unpublished |
| Tomato leaf curl virus | Solanum lycopersicum | Malvastrum Spp. | AJ810357.1 | Arch Virol. 2005, Vol. 150(5), pp 845-67 |
| Malvastrum yellow vein Honghe virus | Malvastrum Spp. | Solanum lycopersicum (Tomato) | KU601620.1 | Unpublished |
| Malvastrum yellow vein Yunnan virus | Malvastrum Spp. | Solanum lycopersicum (Tomato) | KU975394.1 | Unpublished |
| Malvastrum yellow vein Yunnan virus | Malvastrum Spp. | Glycine max (Soybean) | AM156860.1 | Unpublished |
