# Supplementary figures and images for: Sida chlorotic leaf virus: a new recombinant begomovirus found in non-cultivated plants and Cucumis sativus L
Source: PeerJ. 2023 Mar 22;11:e15047. doi: 10.7717/peerj.15047 (PMC10039651; doi:10.7717/peerj.15047)

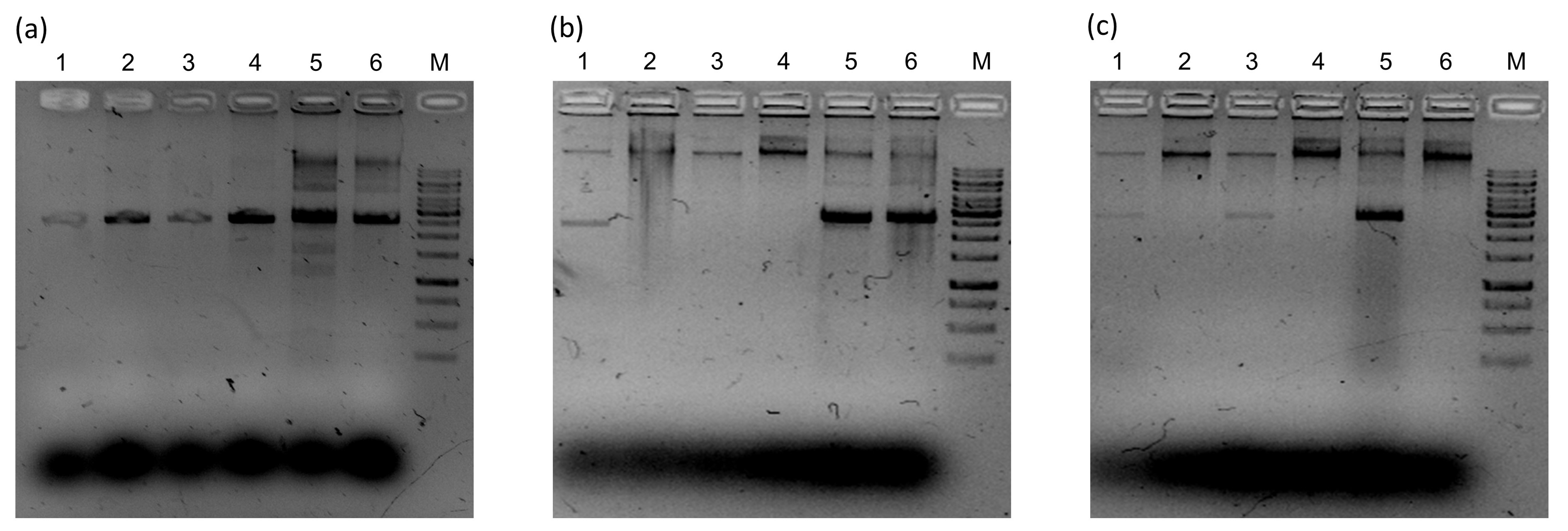

Supplement: Supplemental Information 8 — In all the cases, line “M” correspond to 1Kb DNA ladder (GeneRuler Thermo Scientific), lines 1, 2, 5 and 6 correspond to Sida sp., whereas lines 3 and 4 correspond to Malvastrum sp. RCA products were digested with NdeI (a), XbaI (b) and XhoI (c) for begomovirus detection. [file peerj-11-15047-s008.jpg]

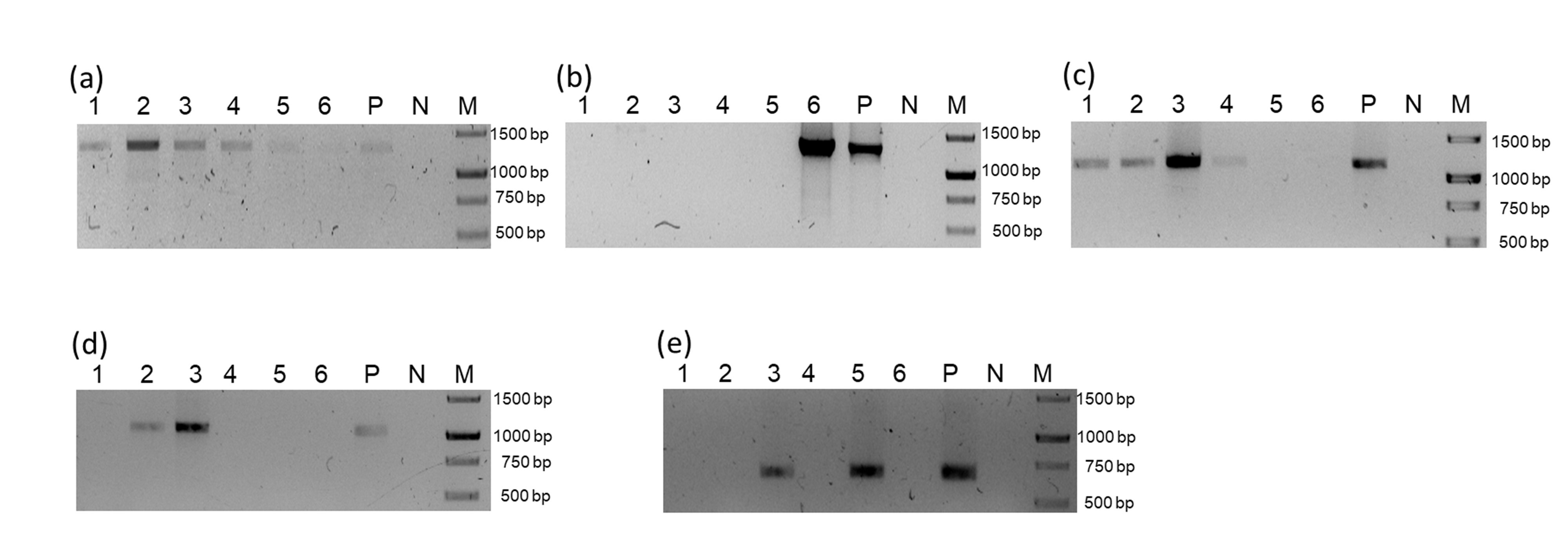

Supplement: Supplemental Information 9 — In all the cases, line “M” correspond to 1Kb DNA ladder (GeneRuler Thermo Scientific), line “P” correspond to positive reaction and line “N” correspond to negative reaction. Lines 1, 2, 5 and 6 correspond to Sida sp., whereas lines 3 and 4 correspond to Malvastrum sp. (A) Detection of SiChLV (F-GAGCACTTCTTCCGTCGATC/R- GCATACACAGGATTAGAGGCA; 1431 bp). (B) Detection of CuChLV (F-AACGTCCTTGGATCACCGA/R-GCAGTGCTAGGTTCATTGTC; 1300 ). (C) Detection of SiMSinV (F-CGGATGTGAGGGTGATGAAG/R-CGAACGATCCTACACAGTGAC; 1131 bp). (D) Detection of OYMMV (F-CTGGTCCTCGTGTACAATGG/R-GATTCTTCGACCTGGTGTCC; 1041 bp). (E) Detection of EuMV (F- CCCACTCTTGCATCTCTTCC /R- GACTCCAGGACTCCACAAAC; 663 bp). [file peerj-11-15047-s009.jpg]

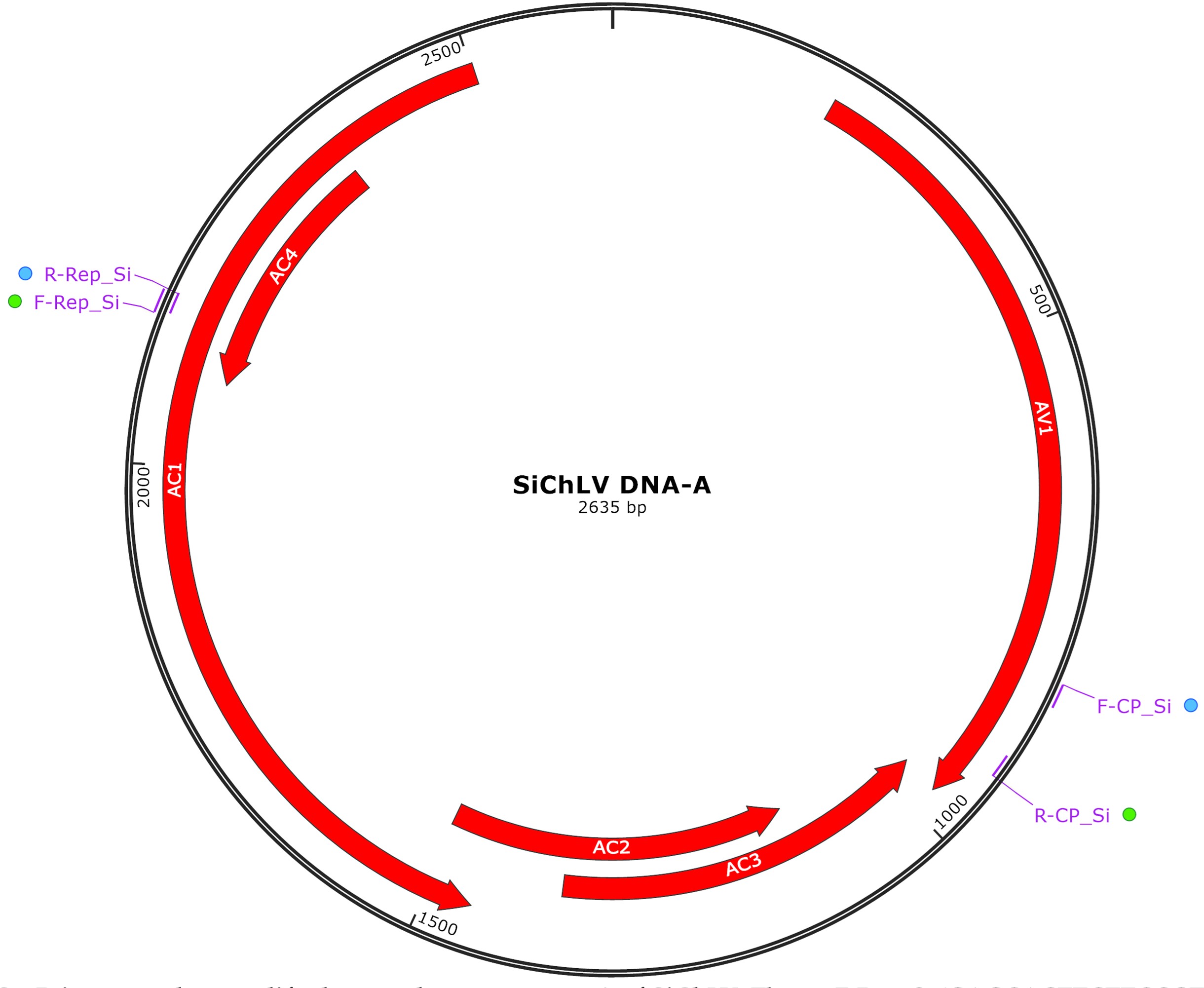

Supplement: Supplemental Information 10 — The set F-Rep_Si (GAGCACTTCTTCCGTCGATC)/R-CP_Si (GCATACACAGGATTAGAGGCA) were used to generate the amplicon denominated “Upper Region (1,431 bp), while the set R-Rep_Si (CCAGATCGACGGAAGAAGTG)/F-CP_Si (TGTCTATAACCACCAGGAAGC) amplified a segment called “Lower Region (1,325 bp). Both PCR products were later used for sequencing via Sanger method. [file peerj-11-15047-s010.jpg]

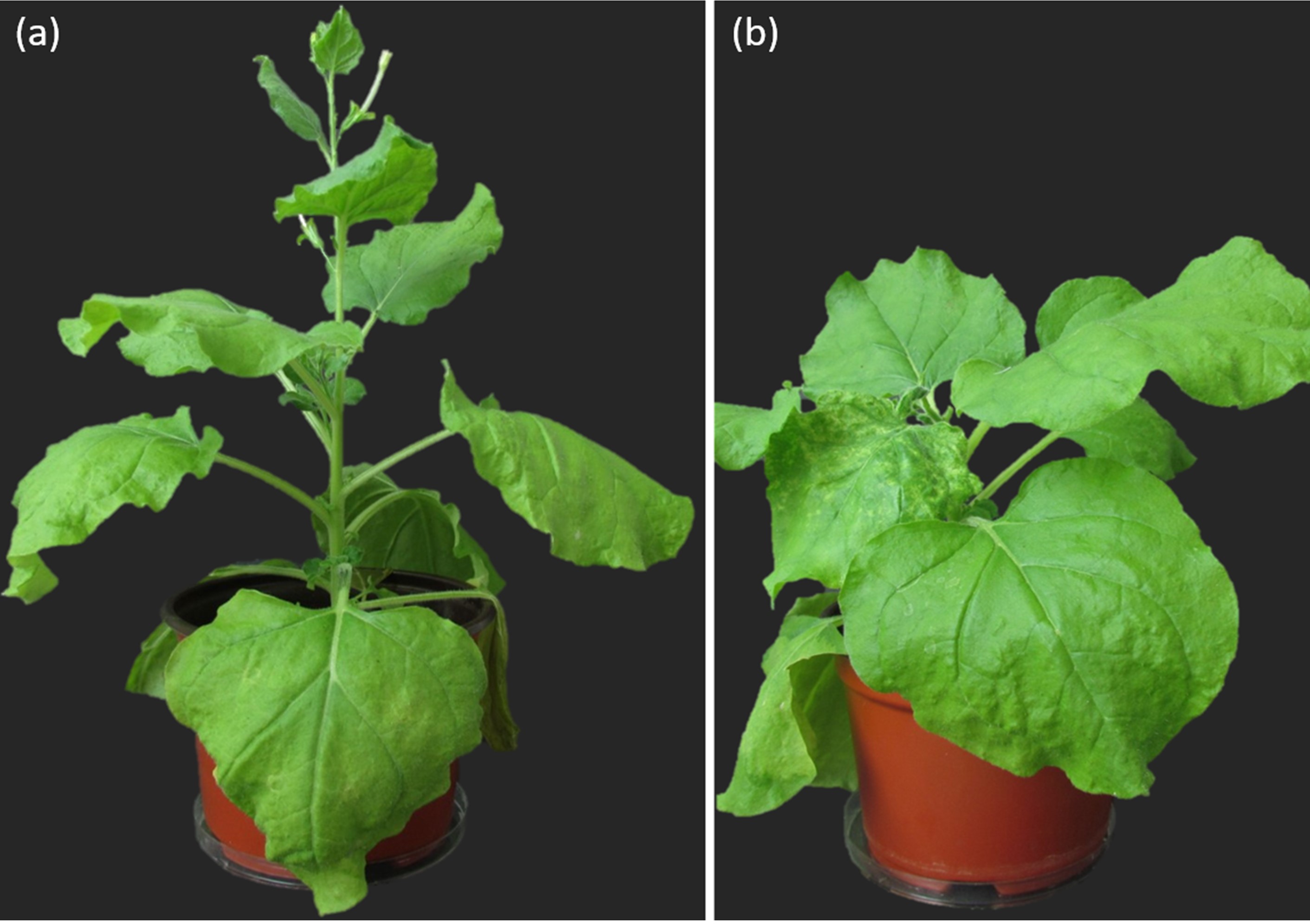

Supplement: Supplemental Information 11 — N. benthamiana plants were agroinoculated with empty pBI121 vector (A) and pBI121-DNA-A/pBI121-DNA-B of SiChLV (B). The photographs were taken 21 days post-infection. [file peerj-11-15047-s011.jpg]

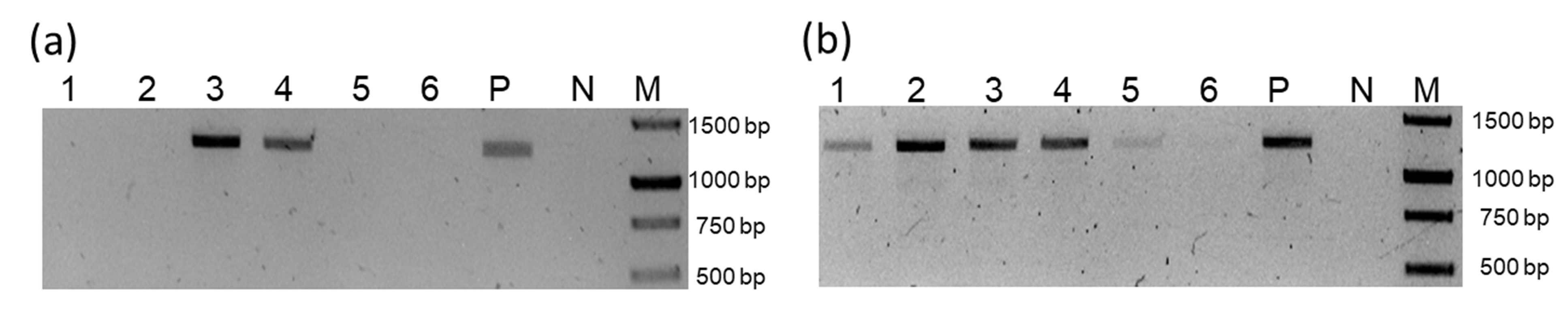

Supplement: Supplemental Information 12 — In all the cases line, “M” correspond to 1Kb DNA ladder (GeneRuler Thermo Scientific), line “P” correspond to positive reaction and the line “N” correspond to negative reaction. (A) Detection of SiChLV (F-GAGCACTTCTTCCGTCGATC/R-GCATACACAGGATTAGAGGCA; 1,431 bp). (B) PCR for CuChLV (F-AACGTCCTTGGATCACCGA/R-GCAGTGCTAGGTTCATTGTC; 1,300 bp). [file peerj-11-15047-s012.jpg]
