# Supplementary material for: Sida chlorotic leaf virus: a new recombinant begomovirus found in non-cultivated plants and Cucumis sativus L
Source: PeerJ. 2023 Mar 22;11:e15047. doi: 10.7717/peerj.15047 (PMC10039651; doi:10.7717/peerj.15047)
Supplement: Supplemental Information 13 [file peerj-11-15047-s013.pdf]

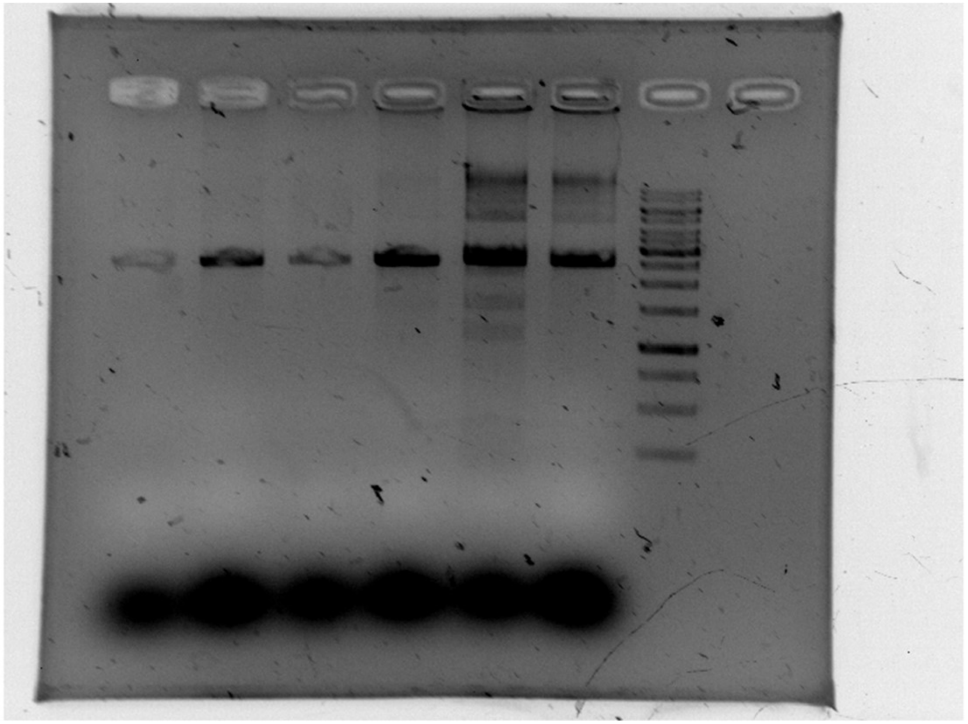

Original figure of Figure S1a

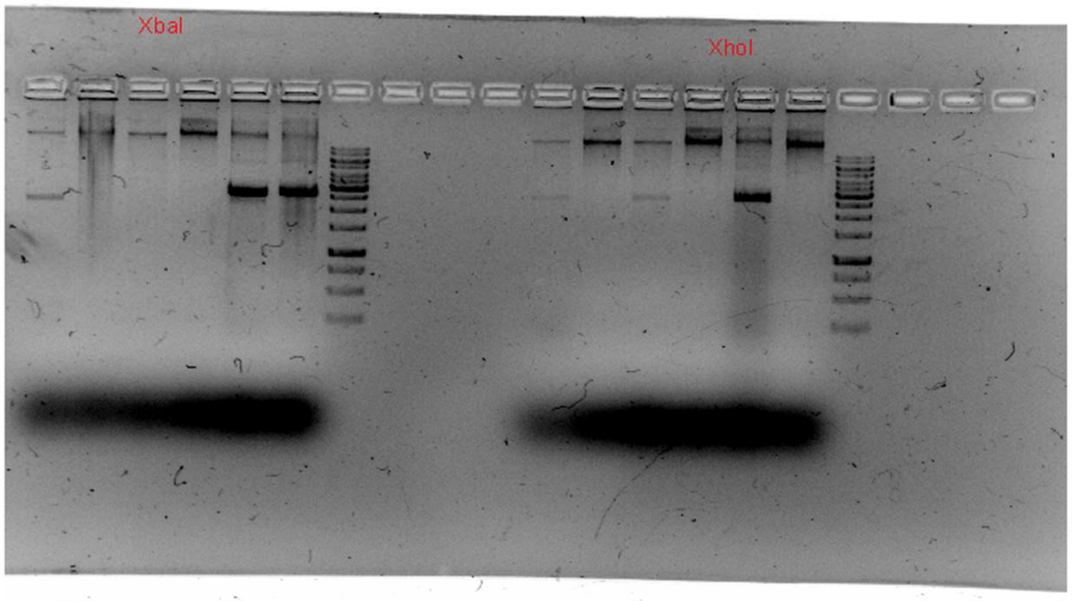

Original figure of Figure S1b y c

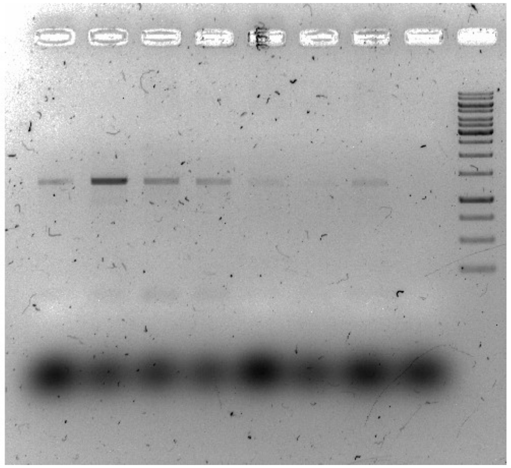

Original figure of Figure S2a

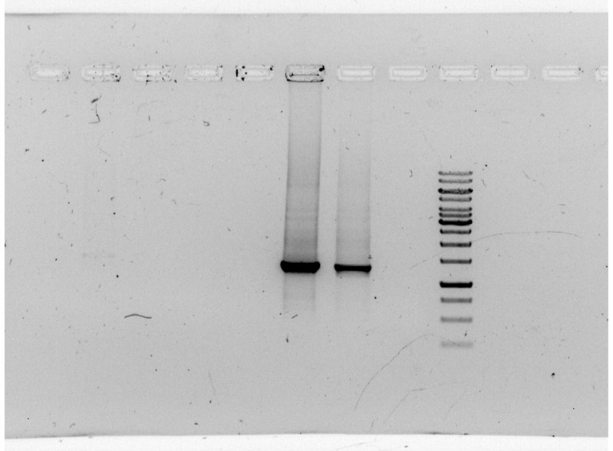

Original figure of Figure S2b

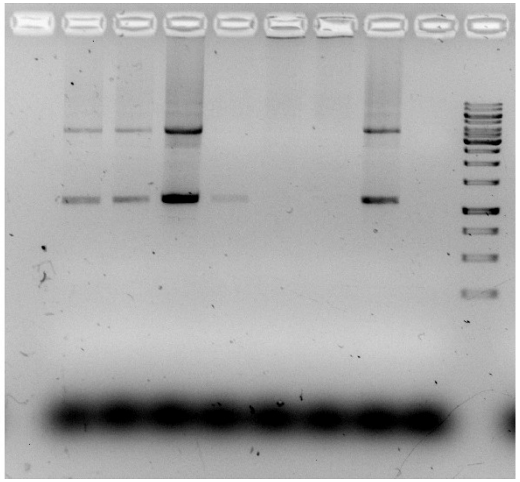

Original figure of Figure S2c

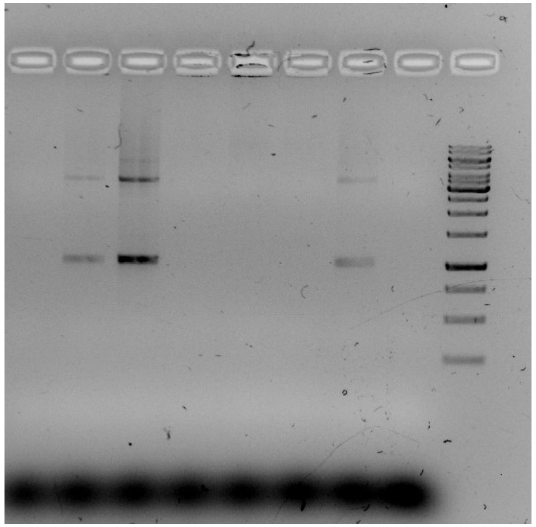

Original figure of Figure S2a

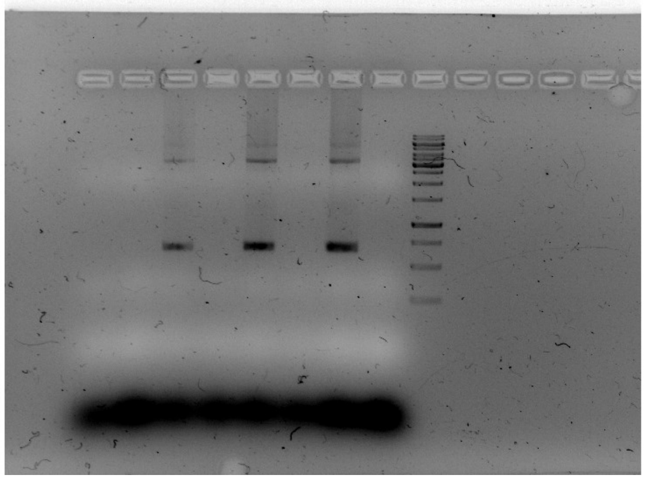

Original figure of Figure S2e

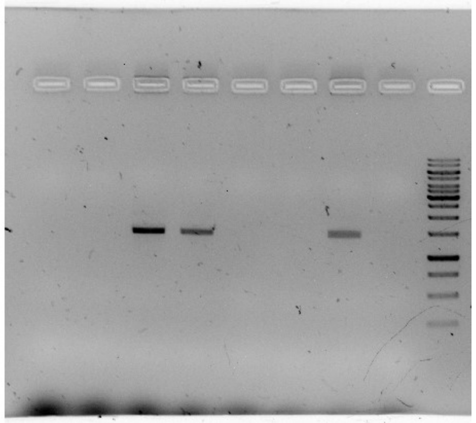

Original figure of Figure S5a

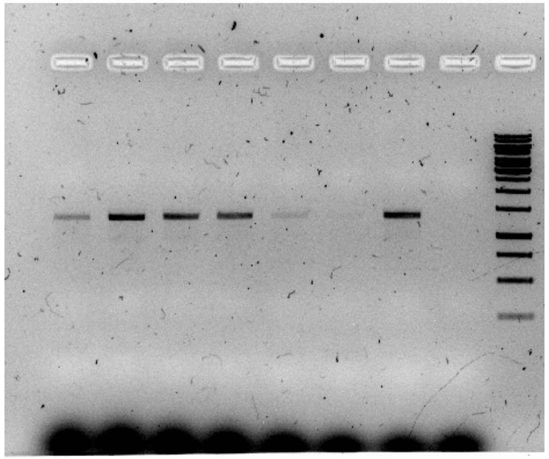

Original figure of Figure S5b
